# Innate Lymphoid Cells in the Central Nervous System

**DOI:** 10.3389/fimmu.2022.837250

**Published:** 2022-02-03

**Authors:** Shuaiwei Wang, Serge A. van de Pavert

**Affiliations:** Aix-Marseille Université, Centre National de la Recherche Scientifique (CNRS), Institut National de la Santé et de la Recherche Médicale (INSERM), Centre d’Immunologie de Marseille-Luminy (CIML), Marseille, France

**Keywords:** NK, ILC1, ILC2, ILC3, ischemic stroke, Alzheimer’s disease, multiple sclerosis, glioma

## Abstract

Immune cells are present within the central nervous system and play important roles in neurological inflammation and disease. As relatively new described immune cell population, Innate Lymphoid Cells are now increasingly recognized within the central nervous system and associated diseases. Innate Lymphoid Cells are generally regarded as tissue resident and early responders, while conversely within the central nervous system at steady-state their presence is limited. This review describes the current understandings on Innate Lymphoid Cells in the central nervous system at steady-state and its borders plus their involvement in major neurological diseases like ischemic stroke, Alzheimer’s disease and Multiple Sclerosis.

## Introduction

**Figure 1:**
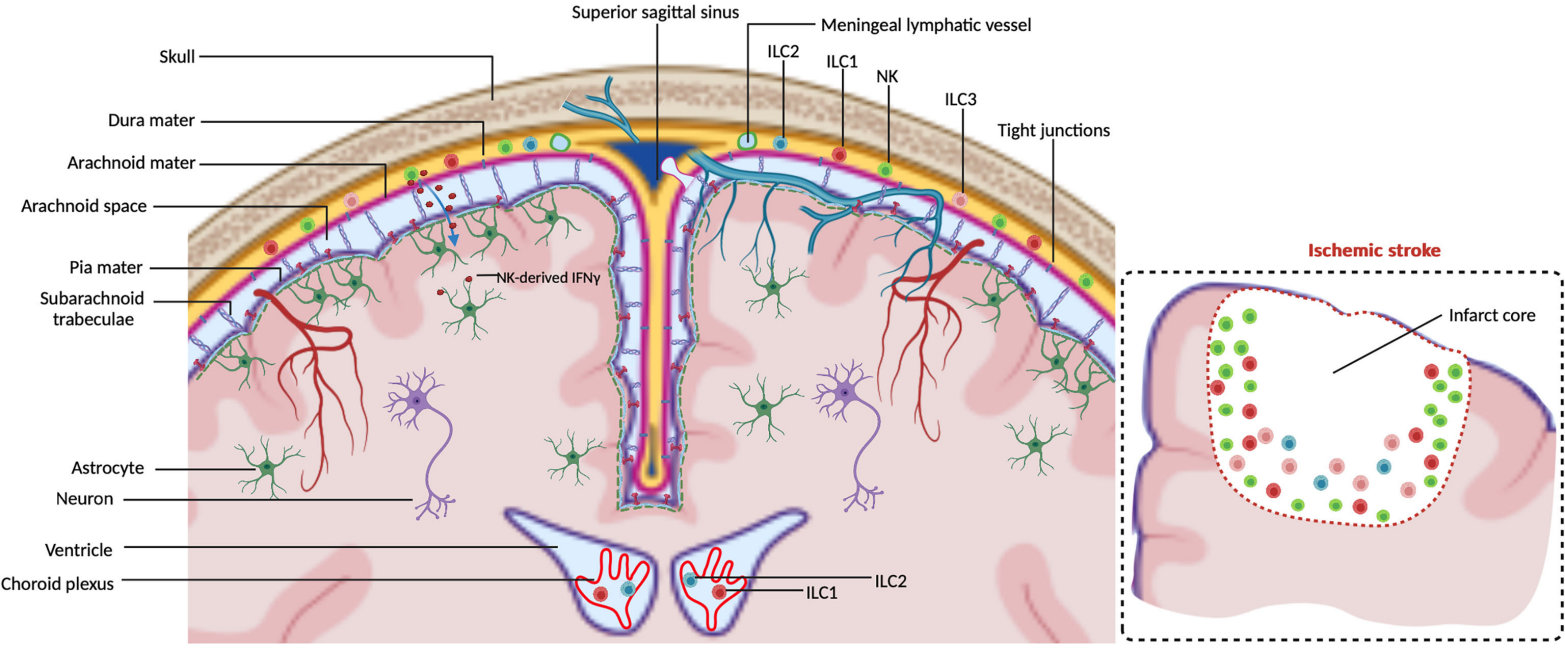
ILCs in the CNS borders at steady-state. Meninges consist of the dura mater, arachnoid mater and pia mater. Dural lymphatic vessels lining the sinuses absorb CSF from the subarachnoid space. Arachnoid- and pia mater are impermeable to immune cells due to tight junctions. NK cells are observed within the meninges to regulate astrocytes by secreting IFN-γ which diffuses into the brain parenchyma. ILC1s are observed to reside in both meninges and choroid plexus. ILC2s and ILC3s are mainly observed within the meninges. The insert illustrates that in an ischemic stroke ILCs accumulate at the lesion border, the majority being NK cells.

The central nervous system (CNS) is a highly sensitive organ and requires protection. Physical protection to the CNS is provided by three meningeal layers. These three layers are the dura mater, adjacent to the skull, the pia mater, located just above the CNS parenchyma, and the arachnoid mater, in between the dura and pia mater (1) (**Figure 1**). Besides the physical protection of the brain, the dura mater layer also harbor a variety of immune cells, whereas arachnoid- and pia mater contain fewer (2). Moreover, dural myeloid and lymphoid cells are replenished from skull or vertebrae bone-marrow in steady-state and inflammatory conditions (3–6). Within the dura mater the superior sagittal sinus and the transverse sinus collect blood from the veins of the brain, meninges and skull and transport this towards the internal jugular veins. The sinuses in the dura mater are the neuro-immunological interface where CNS-derived antigens accumulate and the local antigen presenting cells (APCs) prime patrolling T cells (7). Dural lymphatic vessels lining the sinuses collect CSF from the subarachnoid space and interstitial fluid (ISF) from the brain and drain *via* connections through the nasal-cribriform plate into the nasopharynx lymphatic vasculature (8) towards the mandibular and deep cervical lymph nodes (dcLN) (9, 10). Therefore, the meninges is a critical neuro-immunological interface where immune cells are situated to sense threatening factors such as pathogens and antigens (7). During steady-state, migration of immune cells and macromolecules into the brain parenchyma is restricted by the presence of the blood brain barrier (BBB) around the blood vessels (11), made up of endothelial cells connected by tight junctions (12, 13). Within the CNS, there are immune cells present within the choroid plexus (CP), a villous structure located within brain ventricles comprised of a continuous single layer of epithelium surrounding stroma. The major role of the CP is to produce the cerebral spinal fluid (CSF) (14). Blood vessels with fenestrated endothelium vascularize the CP stroma to enable entry of peripheral leukocytes *via* interactions with blood vessel selectins, integrin ligands and chemokines. Subsequently, recruited leukocytes are able to migrate through the epithelial monolayer into the CSF, mediated by the chemokines in the choroid plexus (15).

A variety of innate and adaptive immune cell subsets including mast cells, dendritic cells (DCs), monocytes, macrophages, T cells and B cells are located in the meninges and CP under steady-state conditions (3, 7, 14, 16–20). The detection of the relative newly described Innate Lymphoid Cells (ILCs) (21) at the CNS borders under steady-state conditions and damaged parenchyma gained increased attention in recent years. ILCs are the innate counterparts of T cells but lack antigen receptor rearrangement. The first ILC subset to be described was the conventional natural killer (cNK) cell in 1975 (22, 23). Later, Lymphoid Tissue Inducer (LTi) cells were described (24), followed by ILC1, ILC2 and ILC3 members (24–32). NK cells are considered to be the innate counterpart of CD8^+^ T lymphocytes. The other ILCs share the characteristics of helper CD4^+^ T cells, and are hence named helper-ILCs. The CD4^+^ helper T cell populations Th1, 2 and 17 share transcription factors and cytokines with their analogous ILC counterparts, respectively ILC1, 2 and 3. As ILCs lack T cell receptor, their activation does not rely on specific antigens and co-stimulation, but rather requires cytokines and signals usually provided by their tissue of residence. Therefore, they are early and immediate responders to a microenvironmental challenge. ILCs distribute to lymphoid and non-lymphoid tissues, including lymph nodes, intestine, liver, lung, skin, uterus and decidua (21, 33). They have been described as tissue-resident, being maintained and expanding locally (34). Other data suggest that a proportion of the ILCs are migratory (35). The CNS parenchyma is almost devoid of ILCs under steady-state condition due to the presence of brain barriers such as the blood-brain barrier (BBB), meningeal barrier, blood-cerebrospinal fluid (CSF) barrier and the ventricular barrier (3, 36). This raises questions on the presence of supposedly tissue resident ILCs within the immune privileged CNS, and what occurs with ILCs upon an inflammation within the CNS. Here, we provide an overview of the ILC presence within the CNS, including the meninges, during steady-state plus their involvement and function in major neurological diseases.

## ILCs in the CNS at Steady-State

### NK and ILC1

NK cells (NKs) and type 1 innate lymphoid cells (ILC1s) commonly express NK1.1 and are defined as Lin^−^CD45^+^NK1.1^+^ NKp46^+^ lymphocytes, with a notable difference in NKs which express transcription factor Eomes and T-bet while in general ILC1s express transcription factor T-bet exclusively (37, 38). Although both NKs and ILC1s produce the principle cytokine IFN-γ, they display different roles. NKs are cytotoxic, whereas ILC1s are generally non-cytotoxic due to the lower expression of perforin and granzyme B production (39).

CD49a and CD49b are used to distinguish NK from ILC1s in some tissues, such as liver, skin and bone marrow, but not in salivary glands (21). Within the CNS, CD45^high^CD3^−^NK1.1^+^ CD49a^−^CD49b^+^ cells are Eomes^+^T-bet^+^, thus NKs. CD45^high^CD3^−^NK1.1^+^CD49a^+^CD49b^−^ cells are Eomes^−^T-bet^+^, thus ILC1s (38). NKs have been described to convert into intermediate ILC1s (intILC1s) by TGFβ, and notably express CD49a, CD49b and Eomes within a tumor microenvironment (40, 41). CD49a^+^CD49b^+^Eomes^+^ intILC1s are also present within the meninges (38). However, about 40% of the CD49a^+^CD49b^+^ intILC1s in the meninges have been described not to express Eomes. Whether the CD49a^+^CD49b^+^Eomes^−^ intILC1s are unique meningeal resident cells and are functionally different remains unknown. Both NKs and ILC1s have been observed within the meninges during steady-state conditions (3, 38, 42, 43) (**Figure 1**). Moreover, these two populations recirculate through peripheral lymphoid tissues (35), raising the question whether NKs and ILC1s within the meninges are migratory or resident. Compared to the NKs in blood, NKs found in the dura mater express higher levels of CD62L and CD27, which are critical for the maturation- and effector- function of NKs (44, 45) and provide a faster and stronger protection against challenges to the CNS. It has been reported that neurons express chemokine CX3CL1 to recruit CX3CR1^+^ NKs to the brain parenchyma, which is associated with a better prognosis against e.g. glioma (46). NKs found in the dura mater also express higher level of CX3CR1 compared to NKs in blood (3), suggesting that NKs patrol the CNS in the homeostatic state and could rapidly be involved in the reaction to pathological conditions. Moreover, meningeal resident NKs are the main contributors for IFN-γ production which transmigrates through the arachnoid and pia mater to induce the death receptor-ligand TRAIL expression in astrocytes. Subsequently, activation of the death receptor on T-cells limits their numbers and inhibits neuroinflammation (42) (**Figure 1**). These findings suggest that regulating the plasticity of NKs in the meninges might be a potential therapy against neurological diseases. Compared to meninges, fewer CD45^+^CD3^−^NK1.1^+^ lymphocytes have been observed within the choroid plexus (CP) at steady state, most of which are ILC1s but not NKs nor intILC1s (38). However, the role of ILC1s in the CP at steady state is currently unknown.

### ILC2

Type 2 innate lymphoid cells (ILC2s) do not only protect against helminth parasites that infect CNS and lead to aggressive neurological diseases (47), but can also promote tissue repair (48). Neurotransmitter receptors such as neuromedin U receptor (NMUR1) and vasoactive intestinal peptide receptor 2 (VIPR2) expressed by ILC2s mediate the crosstalk between the peripheral nervous system (PNS) and ILC2s (49). Neuromedin U (NMU) secreted by neurons positively regulate activation, proliferation and cytokine production of NMUR1^+^ ILC2s (50, 51) to provide a rapid tissue protection against helminth infection. IL-5 produced by immune cells, including ILC2s, promotes release of vasoactive intestinal peptide (VIP) by sensory neurons. In return, VIP stimulates VIPR2^+^ ILC2s to secret IL-5 (52, 53), providing a strong auto-regulatory loop. Neurotransmitter receptor expression by CNS-resident ILC2s has not been reported yet. The transmembrane receptor RET (REarranged during Transfection) tyrosine kinase in ILC2s is activated by glial-derived neutrotrophic factor (GDNF) and induces IL-5 and IL-13 expression (54). Neurons within the CNS express GDNF, and thus the expression of these cytokines in CNS residing ILC2s could be indicative of a neuronal-ILC2 communication (55). ILC2s have been shown mainly within the dural meninges, but not within the leptomeninges (arachnoid mater and pia mater) at steady-state (56, 57) (**Figure 1**). Although few ILC2s have been detected within the choroid plexus in a healthy young brain, an abundance of this population has been found in the aged brain. The increase of ILC2s in the aged brain is probably due to an accumulation of CNS-resident ILC2s since they do not re-enter circulation (56). Transcriptional plasticity analysis show that NK cells and ILC1s could differentiate into ILC2s within the aged brain (58), suggesting that the shift of other ILCs contribute to the ILC2 increase. Interestingly, the ILC2s in the choroid plexus and meninges from aged mice can be divided into three subsets, with different capabilities to proliferate and produce cytokines. ILC2s in the choroid plexus contain more Arg1^+^Il13^+^ ILC2s which mediate type 2 inflammation, whereas the meningeal ILC2s contained more *Csf2* (encoding GM-CSF) expressing ILC2s. Since ILC2-derived GM-CSF induces differentiation of hematopoietic stem and progenitor cells (HSPC) (59, 60), these ILC2s could improve treatment efficacy when transplanting HSPC during neurodegenerative disease treatments. The heterogeneity of ILC2s in the CNS suggests that the distinctive ILC2 subsets only respond to their corresponding stimulation upon a specific inflammation.

### ILC3

Group 3 ILCs share the expression of transcription factor RORγt and are divided into two main populations, the NCR^−^ and the NCR^+^ ILC3s. The NCR^−^ population includes LTi cells which are generated before birth and LTi-like cells generated after birth (61). ILC3s play critical roles before and after birth (61). NCR^−^ ILC3s are essential for the formation of lymph nodes and Peyer’s patches in the embryo, while both NCR^−^ and NCR^+^ ILC3s regulate mucosal immunity. There have been several examples described on neuro-ILC3 crosstalk. Circadian circuits regulate stability of enteric ILC3s that express circadian clock genes (62–64). Disruption of these genes in ILC3s cause impaired microbiome homeostasis and increase susceptibility to inflammatory bowel disease. VIP produced by enteric neurons is recognized as a regulator for enteric VIPR2-expressing ILC3s, even though the results about regulation of VIP on IL-22 production by these ILC3s remain controversial (65–67). Relatively little is known about the presence and roles for ILC3s within the CNS. Heterogeneous ILC3 subsets LTi/LTi-like cells and NCR^+^ ILC3s have been observed in the healthy meninges (68) (**Figure 1**). Although ILC1s and ILC2s are present within the choroid plexus, barely any ILC3s are detected within the CNS (56).

## ILCs in Neurological Diseases

### NK and ILC1

The global burden of neurological diseases is increasing (69). Recent advancements in neuroimmunology indicate that developing immunotherapies against neurological diseases are beneficial in improving clinical treatment. Therefore, a better understanding of the roles for ILCs could benefit development of immunotherapies. We will restrict the discussion on ILCs in major neurological diseases such as cerebrovascular disease ischemic stroke, demyelinating disease multiple sclerosis (MS), Alzheimer’s disease (AD) and glioma.

Stroke is a major cause of disability and death worldwide and classified into ischemic stroke and hemorrhagic stroke. Innate and adaptive immune cells including microglia, neutrophils, monocytes and lymphocytes play multiphasic roles in ischemic stroke and impact the pathogenesis of ischemic brain injury (70–73). NK cells have been detected in the brain parenchyma of stroke patients and mouse models with induced ischemic stroke (43, 74, 75). We observed that the robust accumulation of NK cells in the stroke lesion is caused by progressive migration rather than *in situ* proliferation (43). The main chemotaxis described thus far for controlling migration of NKs towards the lesion are the CX3CL1/CX3CR1 and CXCL12/CXCR4 axis (43, 74). The roles for NK cells in ischemic stroke are contradictionary. To establish the role of NKs, anti-NK1.1 treatment has been frequently used to deplete NKs and ILC1s. However, it is important to note that NK1.1 is expressed on a subset of (ex)ILC3s, which are NKp46^+^T-bet^+^RORyt^+^ (76), and thus anti-NK1.1 treatment can affect this population. However, it has been shown that there are no NK1.1^+^ (ex)ILC3 within the CNS by using the *RORc^GFP^* fate mapping reporter mouse model (38). Moreover, the presence of the RORγt^+^ ILC population is very limited in ischemic stroke brain when compared to the NK cells and ILC1 (43). Therefore, studies using anti-NK1.1 to mediate depletion affect most likely only NKs and ILC1s, but not ILC3s within the CNS. Depletion of NK cells using anti-NK1.1 decreases infarction size and neurological deficits in MCAO (middle cerebral artery occlusion) stroke model (74). However, we have observed that CXCR4^+^ NK cells protect motor behavioral functions in the photothrombotic stroke model by using anti-NK1.1 mediated depletion. Also, blocking migration towards the lesion by *Cxcr4* deletion specifically in NKs and ILC1s protects motor-behavior after stroke ischemic induction (43). The contradiction in the effects of the NKs between these studies can partly be attributed to differences in behavioral test applied. In the study by Gan et al. a less precise Bederson score testing forelimb flexion has been used, which basically measures resistance to lateral push and circling behavior (77). In our study where we have observed a protective effect, we have used beam-walk assay testing foot slips when mice cross an elevated beam to analyze the motor-behavioral deficits. Indeed, using the *Rag1^-/-^* mice, in which all T cells are absent, but not ILCs or NK cells, an improvement of the motor behavior has also been observed in the tMCAO stroke model (78). These findings on the protective nature of NK cells fit with the recent study by Sanmarco et al. reporting that IFNy from NK cells induced TRAIL expression in LAMP^+^ astrocytes to limit the T cell presence and hence prevent inflammation in EAE (42). The protective IFN-γ production by meningeal NK cells, positively regulating the protective role of LAMP^+^TRAIL^+^ astrocytes, has been shown to be induced by the intestinal microbiome (42). Therefore, another explanation for the contradictionary findings is a possible difference in commensal microbes within the intestines of the mice used in the different labs. Intriguingly, clinical studies showed that dysbiosis of gut microbiota has been correlated with the severity of acute ischemic stroke and mice receiving fecal transplantation of ischemic stroke patients with significant dysbiosis develop more severe brain injury (79, 80). To better understand the role of NK cells in the stroke brain, additional studies on how microbiota affect the regulation of NK cells on stroke brain recovery are required.

Multiple sclerosis (MS) is an autoimmune disease of the central nervous system, with a hallmark of nerve fiber demyelination. The pathological role of Th17 cells in MS and its animal model experimental autoimmune encephalomyelitis (EAE) has been described before (81). Anti-NK1.1 mediated depletion of mainly NK and ILC1s suppress Th17-mediated neuroinflammation in EAE (82). Moreover, specifically deletion of NKs and ILC1s using the *Tbx21* ^−/−^ (encoding T-Bet) and *Tbx21^f/f^* NKp46-Cre^+^ model indicate the importance of these cells in the onset of the Th17 mediated inflation as well (83) (**Table 1**). Indeed, several other studies indicate the protective role of NK cells in neuroinflammation, notably in EAE and MS patients (42, 86–89). NKp44 is only expressed on activated NK cells and mediates both activating and inhibitory signals to NK cells (90). NKp44 ligand (NKp44L) is expressed by astrocytes and the interaction of astrocytes with NK cells is mediated by NKp44L-NKp44 interaction. This interaction activates NK cells function and leads to NK mediated astrocyte cell death (91). Therefore, NKs and ILC1s can either inhibit or enhance inflammation in EAE depending on signaling pathways used.

**Table 1:** Overview of ILCs localization in steady-state and neurological diseases.

	NK	ILC1	ILC2	ILC3
Steady-state	Meninges (3, 42, 43)	Meninges and CP (3, 38, 42, 43)	Meninges and CP (56)	Meninges (68)
Stroke	BP (43, 74)	BP (43)	BP (43)	BP (43)
AD	CSF (84)	N.D.	N.D.	N.D.
MS (EAE)	SCP (82, 83) Meninges (83)	BP (38) Meninges (83)	N.D.	BP and SCP (68) Meninges (68)
SCI	N.D.	N.D.	Meninges and SCP (57)	N.D.
Glioma	TME (85)	N.D.	N.D.	N.D.

There are no ILCs found within the brain parenchyma (BP) at steady-state.

AD, Alzheimer’s disease; EAE, Experimental autoimmune encephalomyelitis; SCI, Spinal cord injury; SCP, Spinal cord parenchyma; TME, Tumor microenvironment; N.D., Not Determined.

ILC1s in the CP of the CNS maintain stable expression of IFN-γ and TNF-α in EAE, which could synergistically regulate the levels of IFN-γR and TNF-R1 expressed by the choroid plexus endothelium (38, 92). IFN-γ upregulates a wide array of trafficking molecules expressed by the choroid plexus epithelium, such as vascular cell adhesion molecule 1 (VCAM1), intercellular adhesion molecule 1 (ICAM1) and chemokines (CCL2, CCL5, CXCL9, CXCL10, CX3CL1), which contribute to the trafficking across of CP epithelial barriers by immune cells (92). Thus, ILC1s in the choroid plexus probably act as a gatekeeper for the entry of neuroinflammation-induced immune cells into the CNS.

Alzheimer’s disease (AD) is a neurodegenerative disorder and research on AD focusses on the two well-established hallmarks, amyloid beta (Aβ) plaques and neurofibrillary tangles (NFT) (93, 94). Bioinformatical experimental and clinical studies indicate that the immune system plays an indispensable role in AD pathology (95–98). NK cells have been also reported in the CSF from AD patients (84). However, the role of NK cells in AD patients and the underlying mechanism mediating the migration of NK cells towards the plaques and interaction with the plaques is unknown. Therefore, it remains to be established if NK cells are present in, or near, the plaques, and with which cells they interact. Since IL7Rα is expressed by the majority of ILC1s and hardly on NK cells in the adult, some IL7Rα^+^ NK cells detected in the CSF of Alzheimer’s disease are most likely ILC1s (21, 84). It does not exclude the possibility that the CP-resident ILC1s enter the CSF to patrol the Alzheimer’s brain. In order to distinguish ILC1s from NK cells in the CSF from Alzheimer’s disease in future studies, CD49a and CD49b can be used. CD49a promote the persistence of CD8^+^ T cells within the skin and increases this population after local antigen challenge (99). In analogy to skin CD8^+^ T cells, the CD49a-expressing ILC1s might also protect brain parenchyma from AD and viral or bacterial infections by promoting the persistence of CD8^+^ cells within the CNS.

Glioma is the most prevalent tumor of the CNS with a high mortality rate (100). High heterogeneity of gliomas indicates the complexity of immune landscape within glioma tumor microenvironment (101). The involvement of microglia, macrophage, effector- and regulatory T cells in glioma is described in detail elsewhere (102–104). NK cells are present within the glioma tumor microenvironment (85) and are attracted towards the tumor by neuronal expressed chemokine CX3CL1. The attraction of the CX3CR1^+^ NK cells is associated with a better prognosis in glioma patients (46). The role of NK cells and NK cell immunotherapy against malignant CNS tumors is discussed in detail elsewhere (105). Summarized, activated NK cells are associated with improved prognosis and survival of glioma patients and therefore strategies to enhance NK cell mediated anti-glioma function could improve clinical outcomes.

We propose that NK cells are involved in regulating CNS diseases in a multiphasic manner. NK cells can be activated at the onset of the disease and secret cytokines to regulate its progression. When NK cells arrive at the focal zone, they are capable of directly interacting with some targets such as neurons, microglia and astrocytes. Natural cytotoxicity receptors (NCR) expressed by NK cells recognize a variety of ligands derived from cells, viruses, bacteria and parasites, which affect the activation or inhibition of NK cells (106). Experimental data support the interaction between NK cells and motor neuron (MN) within the CNS, mediated by NCR NKG2D on NK cells, promoting MN degeneration and impairment (107). IL-2-activated NK cells rapidly form synapses with human microglia, mediated by NKG2D and NKp46. This interaction results in killing of the resting microglia and modulate the innate and adaptive immune responses within the CNS (108). Knowledge about ILC1s in neurological diseases is limited since they were previously mis-characterized as closely related conventional NK cells. The recent ILC1 characterization open new areas of investigations into CNS diseases.

### ILC2

Meningeal ILC2 cell numbers increase after spinal cord injury (SCI) (57). Intriguingly, lung-derived ILC2s present within the meninges have been shown beneficial for the recovery after SCI (57), suggesting that they share some characteristics with meningeal ILC2s. ILC2s in other tissues such as lung and gut express the neurotransmitter receptors NMUR1 and VIPR2. Meningeal ILC2s upregulate the gene encoding the receptor for calcitonin gene-related peptide (CGRP) (57), which implies that they are not only activated by cytokine IL-33 but possibly also by CGRP, a neurotransmitter secreted by nociceptive neurons after SCI (**Table 1**). Whether meningeal ILC2s also express other neurotransmitter receptors involved in ILC2s-neuron communication remains to be established. After SCI induction, *in situ* proliferating ILC2s are capable of positively regulating Th2 cell response by IL-13, which could promote axonal regrowth (109, 110).

ILC2s are also detected in the lesion of mouse stroke model (43), meaning that they are potential candidates to regulate the regeneration of affected neurons within the CNS.

Meningeal ILC2 are mainly present within the dural sinuses which have been shown as a critical site for local antigen presentation and immune cell interactions in the CNS (7, 57). The transfer of bone marrow-derived ILC2s into *Cd132* ^−/−^ (IL2Rγ) mouse model induces CNS demyelination upon CNS viral infection, indicating this process is ILC2-dependent (111). However, using a more specific ILC2 knock-out model is required to establish the exact role of ILC2s in demyelination, as in the *Cd132* knock-out also other ILCs are deleted which can potentially bias the conclusion. Demyelination causes a variety of problems including diminished memory, impaired vision, slurred speech and trouble walking. Identifying the cytokines secreted by meningeal ILC2s and the targeted immune cells which promote demyelination after viral infection will be beneficial for understanding the role of ILC2s in CNS diseases and beneficial to use in treatments to inhibit CNS demyelination caused by viral infections.

Female MS patients show symptoms at a younger age and exhibit more severe disease-course than males in general, the reason of which is not fully understood (112, 113). A possible explanation is that testosterone has been shown to increase IL-33 expression which activates ILC2s, induces Th2 responses and involved in limited Th17-dominated demyelination (114). Therefore, increased IL33 levels in males could lead to an increased ILC2 activation and inhibition of MS related symptoms. Also, since ILC2s play a vital role in suppressing tumor growth and metastasis (115), the gender bias in IL-33 secretion could also contribute to differences in glioma incidence and evolution (116, 117).

### ILC3

LTi cells are part of the ILC3 subset and essential for the development of secondary lymphoid organs (SLOs). A critical step in this process is mediated through the lymphotoxin (LT) α1β2 signaling pathway (118), and the cells are involved in formation of some tertiary lymphoid organs (TLOs) (119, 120). LTi cells are attracted by CXCL13 during embryonic lymph node formation (121) and in analogy, increased CXCL13 levels in the CSF of MS patients attract CXCR5^+^ LTi cells towards the CNS (122). Indeed, detection of RORγt^+^CD3^−^ (ILC3) cells in the sub-meningeal B cell follicles suggest the involvement of LTi cells in MS patients (123). Also in relapsing-remitting MS patients, LTi cells have been observed in blood (124) and associate with a specific lesion tomography. On the contrary, in the mouse experimental model for MS, EAE, LTi cells have not been found in TLO’s within the cerebellum parenchyma, but instead Lymphotoxin expressing B-cells have been suggested as inducers of the TLO (125). In another study on EAE, an increase of OX40L^+^ and CD30L^+^ ILC3s in the meninges has been observed, but these are not associated with TLO’s (68). Since LTi cells in adult mice are known to express OX40L and CD30L, these ILC3 within the meninges could very well be LTi cells (126) (**Table 1**). Combined, results from patients and mouse models suggest that the peripheral LTi cells could organize meningeal lymphoid follicles in specific circumstances in MS or EAE, while in other cases their function would be taken over by other cells expressing lymphotoxin.

ILC3s are essential in CNS inflammation, as deletion of MHC-II^+^ ILC3s cells results in loss of symptoms associated with EAE. In this neuroinflammatory model, ILC3’s are capable of presenting antigen to autoimmune T cells in focal lesions and thereby mediate neuroinflammation within the CNS parenchyma (83, 127). Similarly, MHC-II expression by some LTi cells (24, 128) suggests that LTi cells could also promote inflammation in EAE by initiating circulating inflammatory T cells. How antigens are obtained by these ILCs and present it on their MHC-II remains unknown, as they have not been shown to be phagocytic. Accumulated ILC3s, including LTi cells and other ILC3s, are capable of producing pro-inflammatory cytokines such as IFN-γ, IL-17 and GM-CSF, which are responsible for chronic inflammation (68, 83). Besides cytokines, ILC3s could regulate the function and survival of memory CD4^+^ T cells by expressing CD30L and OX40L (129). OX40L expressed by ILC3s is reported to regulate the homeostasis of intestinal Treg cells (130). The existence of Treg cells and ILC3s in the stroke lesion (43, 131) indicates a crosstalk between ILC3s and Treg cells in this disease.

## Concluding Remarks

Although the presence of ILCs in the meninges and choroid plexus in steady-state has been shown, their origin and maintenance remain unknown. Circulating ILC progenitors in the blood might replenish these subsets as was shown in human (132). Previously shown for CNS B-cell and myeloid cell renewal, the contribution of skull and vertebral bone marrow to ILC maintenance has not been investigated (4). Brain barriers prevent the migration of ILC into brain parenchyma at steady-state. It has been shown that neurological diseases cause break down of BBB and meningeal integrity (133–135). The permeability of the BBB is notably increased in stroke and glioma during which the vasculature bed is completely remodeled and re-constructed (136, 137). Also, tight junctions within the BBB are disrupted by molecules such as matrix metalloproteinases (MMPs) in ischemic stroke model (138). Similar as in the BBB, tight junctions in the arachnoid and pia mater might also be disrupted in the inflammatory conditions. These processes could lead to a massive invasion of immune cells, and ILCs, towards the brain lesion. In this model, the ILCs are absent within the brain parenchyma in steady-state conditions but infiltrate the lesion from the dural meninges and local blood vessels upon insult and loss of meningeal- and blood-brain- barrier function.

Considering the importance of ILCs in neurological diseases, such as NK cells in ischemic stroke as well as ILC2s and ILC3s in EAE, knowing the origin and maintenance could aid inducing and culturing these cells *in vitro*. Subsequently, these cells can contribute to developing therapies. The interaction of ILCs with other immune cells such as T cells in the CNS tissues remains to be studied. This knowledge will enhance our understanding of pathological or protective immune responses. Research on ILCs and neuroimmunology has gained much attention in the last few years, whereas the knowledge of ILC-CNS crosstalk remains to be improved. The description of ILC-neuron circuits in peripheral tissues such as lung and intestine (50, 51, 54, 66, 139) raise the question of whether ILCs could also directly communicate with neurons within the CNS.

## Author Contributions

SW and SP wrote the manuscript. SP supervised the project. All authors contributed to the article and approved the submitted version.

## Conflict of Interest

The authors declare that the research was conducted in the absence of any commercial or financial relationships that could be construed as a potential conflict of interest.

## Publisher’s Note

All claims expressed in this article are solely those of the authors and do not necessarily represent those of their affiliated organizations, or those of the publisher, the editors and the reviewers. Any product that may be evaluated in this article, or claim that may be made by its manufacturer, is not guaranteed or endorsed by the publisher.
